# Avian SERPINB12 Expression in the Avian Oviduct Is Regulated by Estrogen and Up-Regulated in Epithelial Cell-Derived Ovarian Carcinomas of Laying Hens

**DOI:** 10.1371/journal.pone.0099792

**Published:** 2014-07-14

**Authors:** Gahee Jo, Whasun Lim, Seung-Min Bae, Fuller W. Bazer, Gwonhwa Song

**Affiliations:** 1 Department of Biotechnology, College of Life Sciences and Biotechnology, Korea University, Seoul, Republic of Korea; 2 Center for Animal Biotechnology and Genomics and Department of Animal Science, Texas A & M University, College Station, Texas, United States of America; Ludwig-Maximilians University, Germany

## Abstract

Serine protease inhibitors (SERPINs) are involved in a variety of biological processes such as blood clotting, angiogenesis, immune system, and embryogenesis. Although, of these, SERPINB12 is identified as the latest member of clade B in humans, little is known of it in chickens. Thus, in this study, we investigated SERPINB12 expression profiles in various tissues of chickens and focused on effects of steroid hormone regulation of its expression. In the chicken oviduct, SERPINB12 mRNA and protein are abundant in the luminal (LE) and glandular (GE) epithelia of the magnum in response to endogenous or exogenous estrogen. Furthermore, SERPINB12 mRNA and protein increase significantly in GE of cancerous ovaries of laying hens with epithelia-derived ovarian cancer. Collectively, these results indicate that SERPINB12 is a novel estrogen-stimulated gene that is up-regulated by estrogen in epithelial cells of the chicken oviduct and that it is a potential biomarker for early detection of ovarian carcinomas in laying hens and women.

## Introduction

The chicken oviduct is well known as an excellent model for basic mechanisms of organogenesis during developmental events. In addition, the oviduct of laying hens consists of four anatomical and functional components: the infundibulum is the site of fertilization of the ovum; the magnum produces components of egg-white; the isthmus for provides for formation of the shell membrane in eggs; and the shell gland supports formation of the egg shell [1], [2]. Furthermore, the chicken oviduct is very suitable for investigating morphological and physiological effects on development and differentiation of reproductive tissues and organs of vertebrates in response to sex steroid hormones [3]. Estrogen is the primary sex steroid hormone that plays pivotal roles in cell survival and proliferation, synthesis and secretion of egg white proteins and formation and differentiation of tubular glands in the developing oviduct of laying hens [4]. Results of our previous studies indicate that estrogen-stimulated genes such as *AHCYL1, AvBD11, A2M, CTNNB1, ERBB1, PTN*, *SERPINB3, SERPINB11*, and *SPP1* are involved in development of the chicken oviduct due to their activation of various regulatory mechanisms that are mediated via diverse trans-activating transcription factors and complex actions of estrogen to effect physiological changes [5], [6], [7], [8], [9], [10], [11], [12], [13]. Moreover, changes in circulating concentrations of estrogen following cessation and reinitiation of ovarian steroidogenesis in response to induced molting regulates temporal patterns of expression of these genes during remodeling and development of the oviduct in laying hens [14]. However, identification and characterization of additional genes to elucidate mechanisms responsible for development of the chicken oviduct is clearly warranted.

Serpins (serine protease inhibitors) are the largest family of protease inhibitors present in blood and involved in various proteolytic cascades including blood clotting and the complement system in extracellular fluid and blood [15]. A subset of serpins (clade B serpins) are called human ov-serpins because their molecular structures are very similar to egg white proteins such as ovalbumin [16]. Clade B serpins reside primarily within cells and play important roles in various biological processes including protection against certain apoptotic cascades, inhibition of cell migration, and tumor suppression [17]. Of these, *SERPINB12*, also known as yokopin, is located at 18q21.3 in the human genome encoding a protein of 405 amino acids (about 46 kDa) and widely expressed in various human tissues such as brain, heart, lung, liver, spleen, pancreas, kidney, testes, ovary and placenta [18]. However, little is known about the expression and hormonal regulation of SERPINB12 in chickens.

Our research has discovered candidate genes and their expression in the oviduct of immature chicks treated with diethylstilbestrol (DES, a synthetic estrogen agonist) [19]. We have also used DNA microarrays to identify estrogen regulated genes critical to regulatory mechanisms for regression and recrudescence of the oviduct of laying hens during induced molting [14]. Our gene profiling data showed that SERPINB12 increased in the oviduct of chicks treated with DES, but not non-treated chicks; therefore, it is likely to be regulated by changes in secretion of estrogen from the ovary during regression and recrudescence of the oviduct of laying hens during the molting process. Therefore, we hypothesized that SERPINB12 has essential roles in development and differentiation of the oviduct in response to estrogen in laying hens. Hence, the aims of this study were to: 1) determine gene expression patterns for SERPINB12 in the immature chick oviduct treated with estrogen; 2) determine whether expression of SERPINB12 changes in the oviduct of laying hens during regression, remodeling and recrudescence phases following induced molting; and 3) compare differential expression patterns for SERPINB12 in normal and cancerous ovaries of laying hens.

## Results

### SERPINB12 mRNA expression in various organs from chickens (Study one)

**Figure 1:**
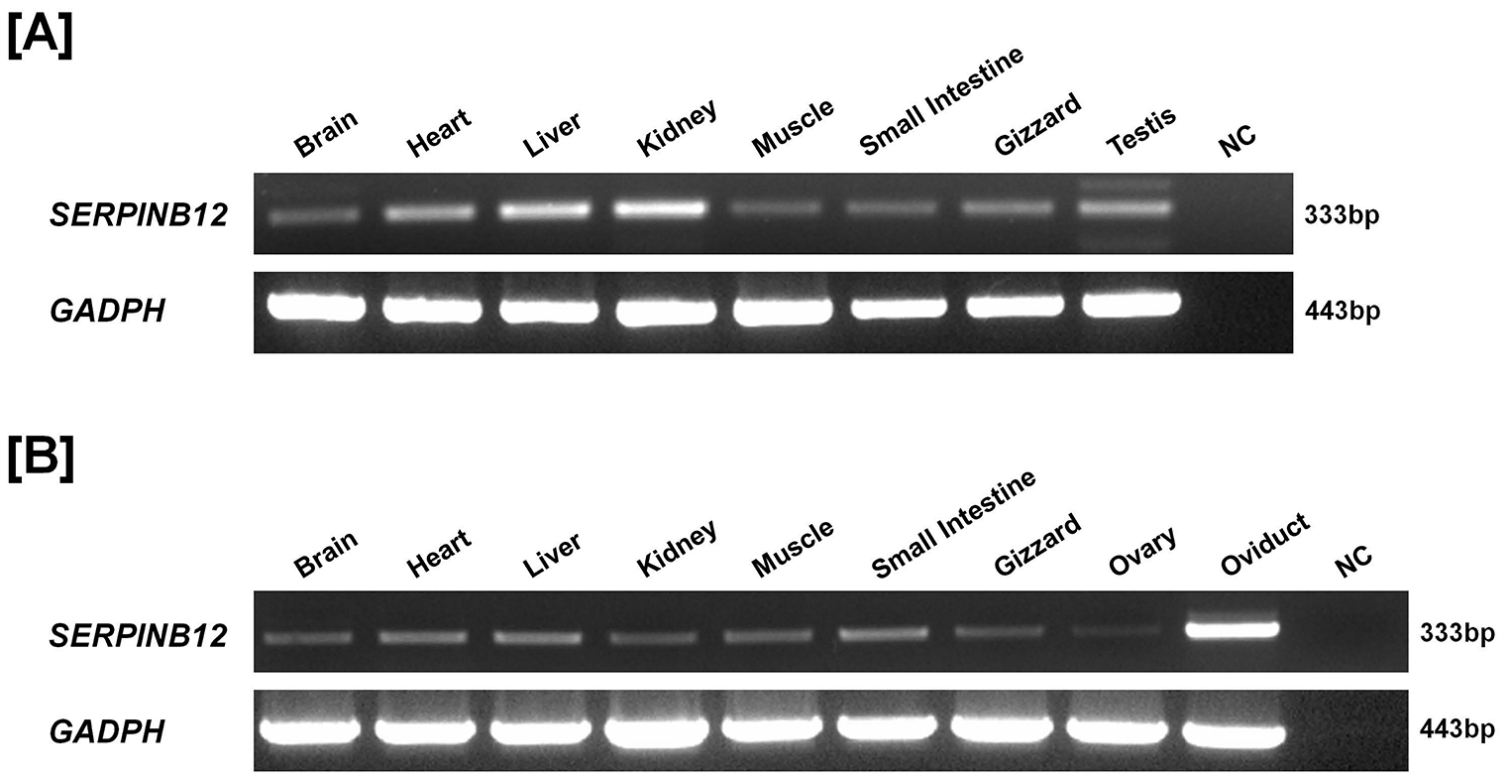
Expression of SERPINB12 in various organs of both male and female chickens. Results of RT-PCR analysis using cDNA templates from different organs of both male [A] and female [B] chickens with chicken SERPINB12 and chicken GAPDH-specific primers are shown. SERPINB12 gene was generally expressed in all organs from male and female chickens. Its expression was highly abundant in the chicken oviduct.

To determine tissue-specific patterns of expression of *SERPINB12* mRNA in chickens, RT-PCR analyses were performed using various organs including brain, heart, liver, kidney, muscle, small intestine, gizzard, testis, ovary, and oviduct of 1- to 2- year-old males (n = 3) and females (n = 3). As shown in Figure 1A and 1B, *SERPINB12* gene is moderately expressed in all organs irrespective of sex. However, there was high expression of *SERPINB12* mRNA in the oviduct of female chickens and in liver and kidney from males. These results are very similar to a previous report that SERPINB12 is detected in many adult and fetal tissues of humans [18]. These results suggest that SERPINB12 plays an important role(s) in various organs.

### Estrogen regulates SERPINB12 expression in the developing chicken oviduct (Study two)

**Figure 2:**
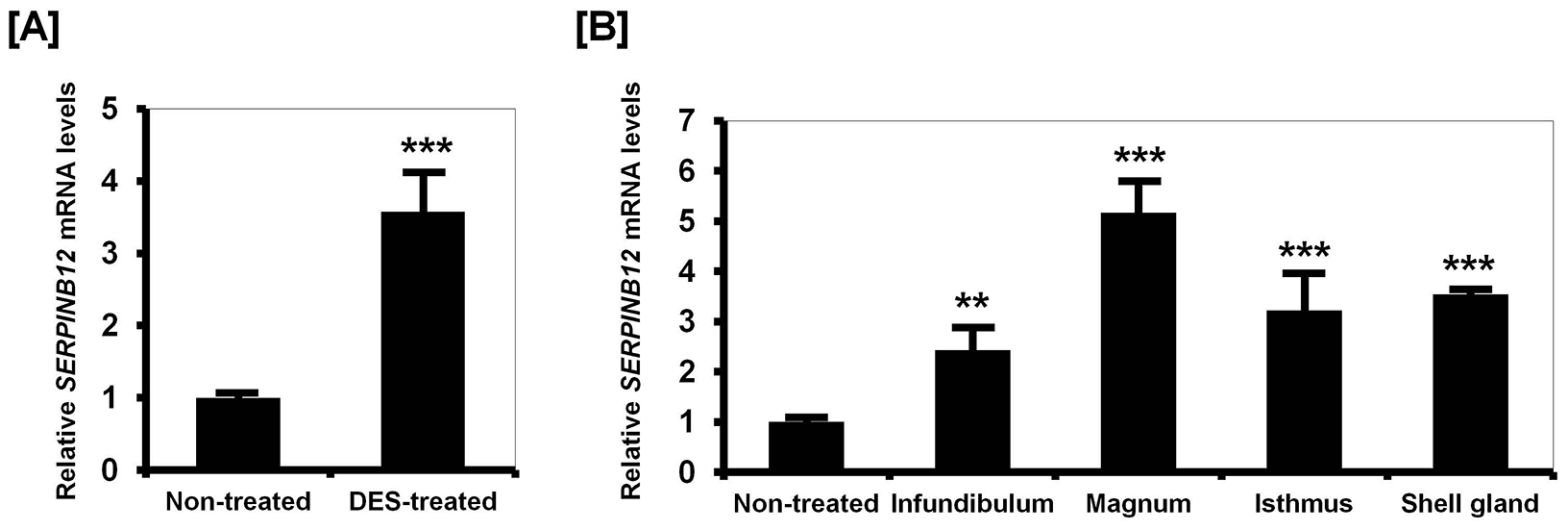
Effect of DES on tissue specificity of expression of chicken SERPINB12 by quantitative PCR analyses. Quantitative PCR analyses showed that *SERPINB12* mRNA was more abundant in the oviducts of DES-treated chicks, as compared with oviducts from control chicks [A]. Among the four segments of oviducts from DES-treated chicks, SERPINB12 was most highly expressed in the magnum [B]. The asterisks denote statistically significant differences (mean ± SEM; ** *P*<0.01 or *** *P*<0.001).

**Figure 3:**
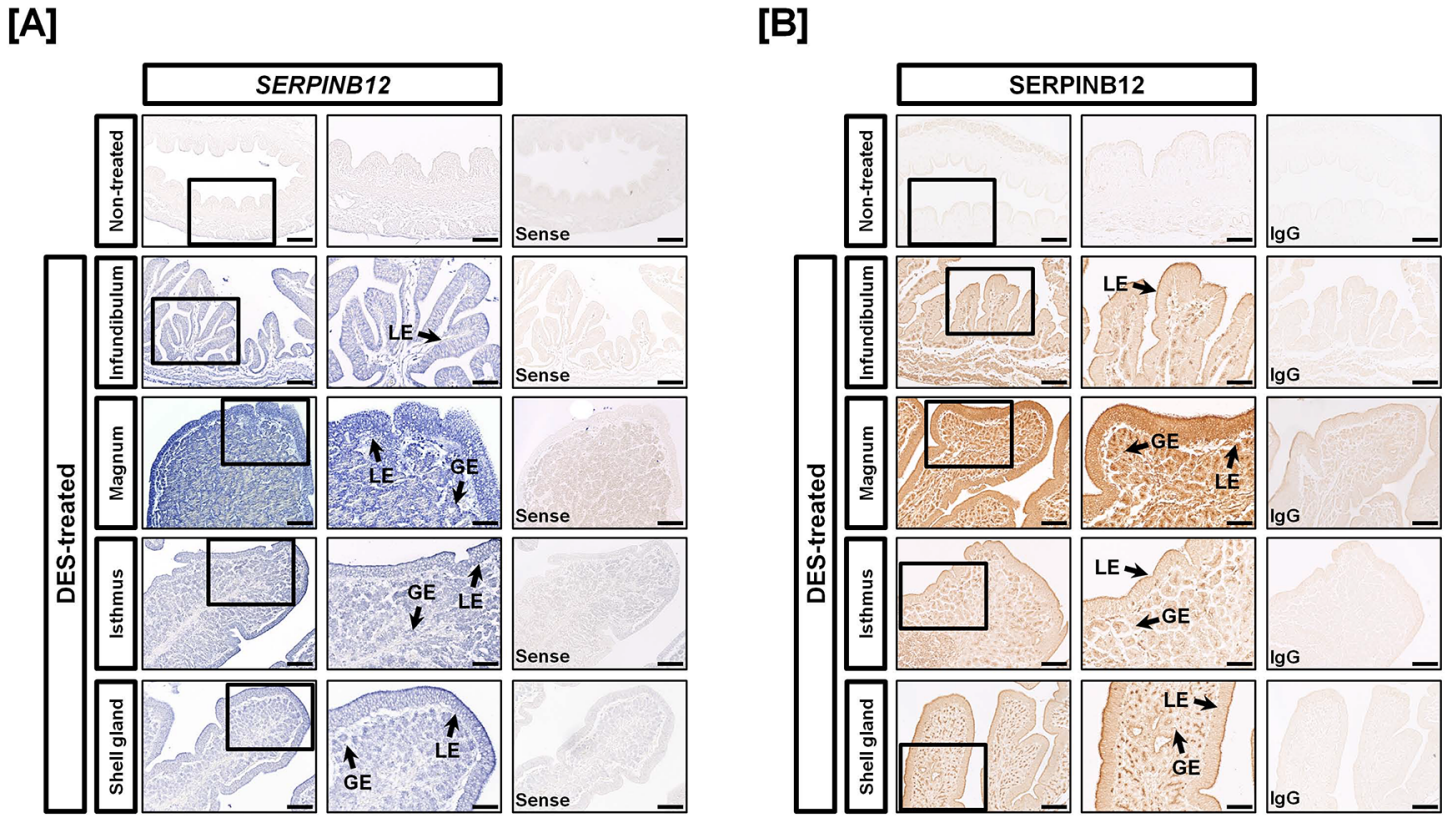
Localization of SERPINB12 mRNA and protein in oviducts of DES-treated and non-treated chicks. [A] *In situ* hybridization analyses indicated cell-specific expression of *SERPINB12* mRNA in GE and LE of the four segments of oviducts from DES-treated chicks. However, there was no expression of *SERPINB12* in oviducts from control chicks. [B] Immunoreactive SERPINB12 protein was localized to LE and GE of oviducts of chicks treated with DES, especially the magnum. For the IgG control, normal rabbit IgG was substituted for the primary antibody. Sections were not counterstained. Legend: LE, luminal epithelium; GE, glandular epithelium; *Scale bar* represents 200 µm (the first columnar panels, sense and IgG) and 50 µm (the second columnar panels).

To investigate the effects of estrogen on development of the chick oviduct, we first performed quantitative PCR analysis using each segment of the neonatal female chick oviduct treated with DES: infundibulum, magnum, isthmus and shell gland. As illustrated in Figure 2A, *SERPINB12* mRNA increased 3.58-fold (*P*<0.001) in the oviduct of DES-treated chick as compared to non-treated chicks. *SERPINB12* mRNA increased 2.44- (*P*<0.01), 5.17- (*P*<0.001), 3.22- (*P*<0.01) and 3.54-fold (*P*<0.001) in the infundibulum, magnum, isthmus and shell gland segments of the chick oviduct, respectively, in response to DES (Figure 2B). In addition, *in situ* hybridization analysis revealed that *SERPINB12* mRNA was expressed abundantly by luminal (LE) and glandular (GE) epithelia of the magnum and moderately expressed by the infundibulum, isthmus, and shell gland of oviducts from chicks treated with DES (Figure 3A). Consistent with these results, immunoreactive SERPINB12 protein was abundant in LE and GE of the magnum and also expressed in other sections of the oviduct at a low level (Figure 3B). However, the non-specific rabbit IgG, used as a negative control, did not detect immunoreactive SERPINB12 protein in the DES-treated chick oviduct.

### Estrogen regulates SERPINB12 expression in the chicken oviduct during the induced molting (Study three)

**Figure 4:**
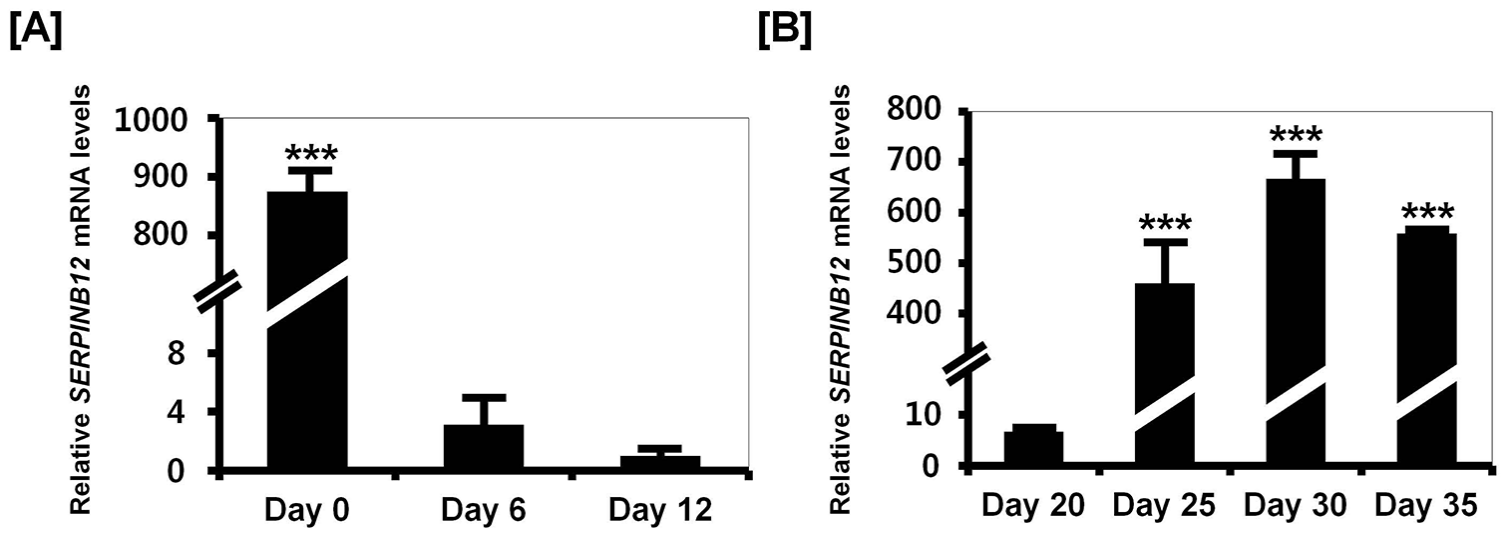
Quantitation of SERPINB12 mRNA during regression and recrudescence of the magnum of laying hens during induced molting. Quantitative PCR was conducted using cDNA templates from the magnum of hens fed a control diet (Day 0), zinc fed hens in which the oviduct was regressing (Day 6 and 12) [A], and hens in which the oviduct was undergoing recrudescence (Day 20, 25, 30, and 35) [B]. These experiments were performed in triplicate and normalized to control *GAPDH* expression. The asterisks denote statistically significant differences (mean ± SEM; ** *P*<0.01 or *** *P*<0.001).

**Figure 5:**
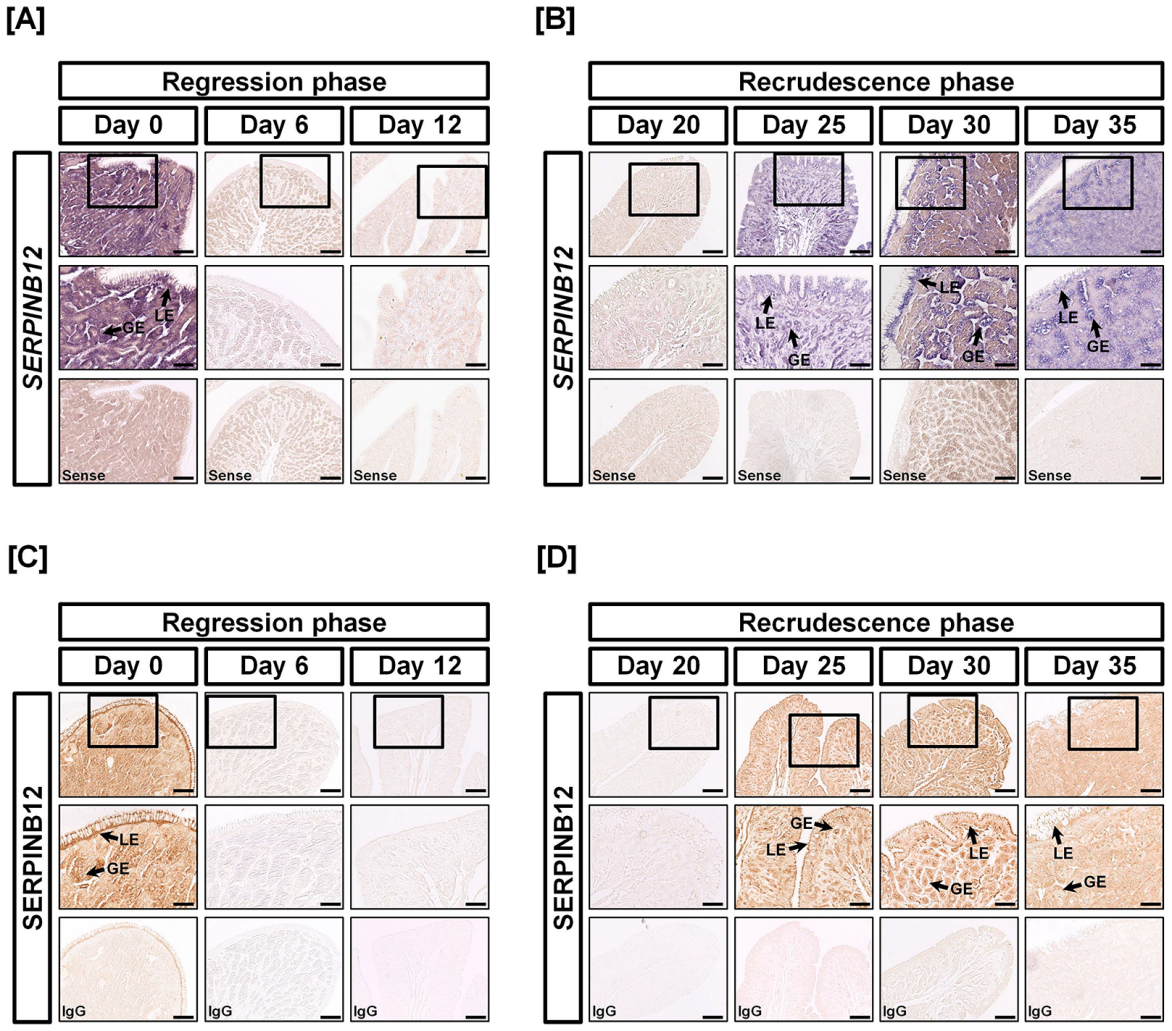
Cell-specific localization of SERPINB12 mRNA and protein during regression and recrudescence of the magnum of oviducts from hens during induced molting. The localization of *SERPINB12* mRNA in the magnum of hens on different days of the regression phase [A] and recrudescence phase [B] was analyzed by *in situ* hybridization. It was predominantly expressed in GE and LE of the magnum on Days 0, 25, 30 and 35 of the recrudescence phase of molting. On the other hand, the expression of SERPINB12 was rarely detected on Days 6, 12 and 20 of the regression phase of dietary zinc induced molting. Immunoreactive SERPINB12 protein in the magnum during the regression phase [C] and recrudescence phase [D] was analyzed by immunohistochemistry. Consistent with *SERPINB12* mRNA localization, SEPRINB12 protein was mainly detected in GE and LE of the magnum on Days 0, 25, 30 and 35 during the recrudescence phase of the oviduct during molting. Legend: LE, luminal epithelium; GE, glandular epithelium; *Scale bar* represents 200 µm (the first horizontal panels, sense and IgG) and 50 µm (the second horizontal panels).

In this study, we evaluated the effects of estrogen on expression of SERPINB12 during the regression and recrudescence phases of molting in laying hens. As shown in Figure 4A, SERPINB12 expression decreased sharply between Days 6 and 12 of molting (regression phase) and the increased 6.75-fold and 666.83-fold (*P*<0.01∼0.001) between Days 20 and 30, respectively, of the molting process (recrudescence phase). *In situ* hybridization analysis revealed that *SERPINB12* mRNA is expressed in the LE and GE on Day 0, but expression ceased as concentrations of estrogen in blood decreased from Days 1 to 20 of the molting period (Figure 5A and 5B). However, expression of *SERPINB12* mRNA increased with increasing concentrations of estrogen in blood between Days 21 and 35 of the molting process. Likewise, immunohistochemial analyses revealed that SERPINB12 protein was predominantly localized to LE and GE of the oviduct during the regeneration phase of the oviduct following induced molting.

### Distinct expression pattern of SERPINB12 in normal and cancerous ovaries of hens (Study four)

**Figure 6:**
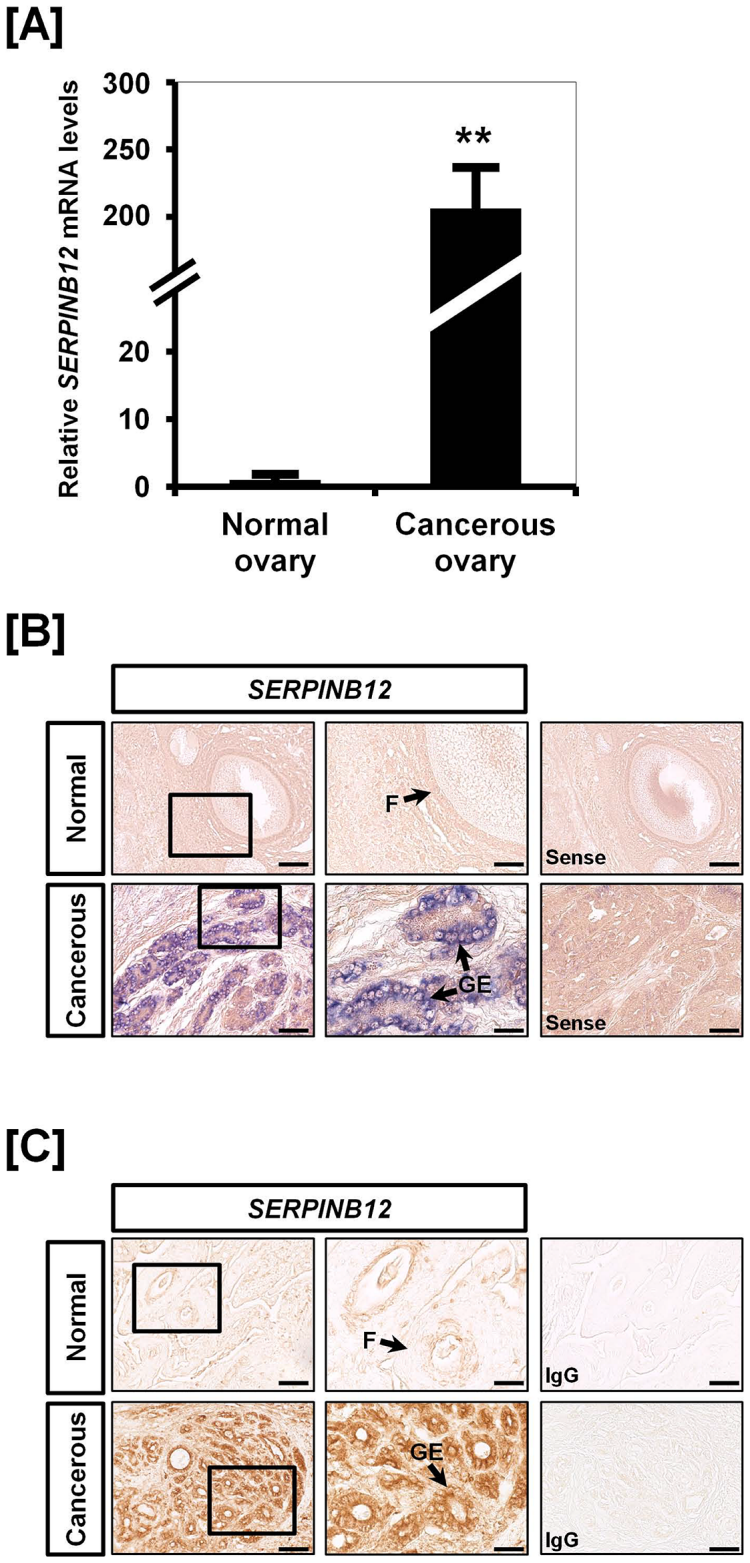
Distribution and localization of SERPINB12 mRNA and protein in normal and cancerous ovaries from laying hens. [A] Quantitative PCR was conducted using cDNA templates from normal and cancerous ovaries of hens. The expression of *SERPINB12* was unique to cancerous ovaries of laying hens as compared with normal ovaries from laying hens. [B] *In situ* hybridization analyses indicated cell-specific localization of *SERPINB12* mRNA in normal and cancerous ovaries from laying hens. *SERPINB12* was strongly expressed in the GE of cancerous ovaries whereas there was no expression in any cells of normal ovaries. [C] Immunoreactive SERPINB12 protein, as for *SERPINB12* mRNA, was also unique to the GE of cancerous ovaries from laying hens. Legend: F, follicle; GE, glandular epithelium. *Scale bar* represents 50 µm (the first columnar panels, sense and IgG) and 200 µm (the second columnar panels).

We previously reported that *estrogen receptor alpha (ESR1)* mRNA expression increased about 51-fold (*P*<0.001) and immunoreactive ESR1 protein was most abundant in GE of cancerous ovaries, but not detectable in normal ovaries. Those results indicated that estrogen-stimulated genes in the oviduct may also increase in cancerous ovaries of hens in response to estrogen. Thus, we examined whether SERPINB12 is expressed in cancerous ovaries of laying hen in the present study. As illustrated in Figure 6A, *SERPINB12* mRNA expression increased 206.16-fold (*P*<0.01) in cancerous as compared to normal ovaries of laying hens. To compare cell-specific localization of *SERPINB12* between normal and cancerous ovaries, we performed *in situ* hybridization and immunohistochemical analyses to find abundant expression of SERPINB12 mRNA and protein specifically in GE of cancerous ovaries, but not stromal cells or blood vessels of cancerous ovaries, or in any cells of normal ovaries (Figures 6B and 6C). This result indicates that SERPINB12 expression increases in cancerous ovaries of laying hens as compared with normal ovaries, and that its expression is co-localized to cells in cancerous ovaries expressing ESR1.

## Discussion

Results of the present study provide the first evidence of *SERPINB12* gene expression in the reproductive organs of female chickens in response to estrogen. Additionally, the results are the first to demonstrate that SERPINB12 expression is uniquely expressed in cancerous ovaries, but not normal ovaries of laying hens. Thus, results of the present study support our hypothesis that SERPINB12 serves essential roles in development and differentiation of the oviduct, as well as ovarian tumorigenesis in response to estrogen in laying hens.

The serpin superfamily has 16 clades and over 500 members with most involved in proteolytic activity in various biological processes [20]. Of these, the clade B serpins, also known as ovalbumin-related serpins (ov-serpins), are encoded for by 13 genes including *monocyte neutrophil elastase inhibitor (SERPINB1), plasminogen activator inhibitor-2 (SERPINB2), maspin (SERPINB5)*, and *plasminogen activator inhibitor-6 (SERPINB6)* [21]. Because SERPINB12 is the latest known member of clade B, little is known about it or its role in the chicken. In this study, we found that chicken SERPINB12 is expressed moderately in various tissues from both sexes as reported for humans [18]. Interestingly, SERPINB12 mRNA and protein are expressed abundantly in the chicken oviduct, particularly in the LE and GE of the magnum. However, it is also moderately expressed in the infundibulum, isthmus, and shell gland of the chicken oviduct. These results suggest that SERPINB12 plays an essential role in secretion or storage of egg white proteins in the magnum of the chicken oviduct.

Previous gene profiling data indicated that SERPINB12 might be regulated by estrogen in the oviduct of laying hens fed a high zinc diet to induce molting [14]. During molting, the oviduct and ovary of laying hens undergo regression and remodeling and then both undergo recrudescence in response to increases in circulating concentrations of estrogen [22]. Our previous study showed that concentrations of estradiol in serum decreased during the induced molting period (Days 0 to 20) and then increased during the recrudescence period (Days 20 to 35). This result suggested that proliferation of oviductal cells between Days 25 and 30 of the molting period is likely induced by estrogen following resumption of ovarian steroidogenesis. Based on those results and our gene profile data, we selected and examined SERPINB12 expression in the current study, and we found it to increase significantly in oviductal tissue during the remodeling and recrudescent phases (Day 25 to Day 35) when concentrations of estrogen increase during induced molting. These results indicate that *SERPINB12* is a novel gene likely involved in regulatory mechanisms(s) for differentiation of the oviduct of neonatal chicks, as well as tissue regeneration and remodeling of the oviduct of laying hens in response to estrogen.

It is well know that DES implants and endogenous estrogen induce development of the neonatal chick oviduct including differentiation of oviductal tubular glands and ciliated cells, and the synthesis and secretion of egg white proteins by epithelial cells of the magnum [1], [23]. Our previous studies showed that SERPINB3 and SERPINB11 are also expressed in a cell-specific manner in the chicken oviduct in response to estrogen [5], [8]. Furthermore, using microarray analysis to examine transcript changes in chick oviducts after exposure to DES, we reported that *SERPINB12* mRNA expression changed significantly during oviduct development in chickens [19]. Therefore, we examined expression of SERPINB12 in the oviduct of DES treated chicks in this study and found that expression of SERPINB12 mRNA and protein is also regulated by estrogen in a tissue- and cell-specific manner during development and differentiation of the chicken oviduct. These results suggest that SERPINB12 may have effects on the process of secretion or storage of egg white proteins.

Recently, Crum and colleagues reported that ovarian cancer (pelvic serous cancer) may originate from the distal end of the oviduct [24]. In fact, epithelial cell-derived ovarian cancer (EOC) is one of the most lethal causes of cancer-related deaths among women due to the fact that there are no or few symptoms and lack of markers for its diagnosis in early stages of the disease [25]. Although several animal models for research to develop novel biomarker for early detection of EOC and appropriate medical treatment have been developed, most of them are not effective. Interestingly, laying hens are being recognized as a good model because chicken EOC occurs spontaneously, and tumor progression, and histological and pathological types and stages are very similar to that in women [26], [27]. Trevino and colleagues reported that EOC may arise from the oviduct and about one-half of up-regulated genes in EOC are oviduct-related genes in response to estrogen stimulation in the laying hen model [28]. Indeed, we reported on several estrogen-stimulated genes including *CTSB*, *SERPINB11*, *SERPINB3*, *AHCYL1*, *SPP1*, *A2M*, *PTN*, *AvBD-11, CTNNB1* [6], [7], [9], [12], [29], [30], [31], [32] and several cell cycle genes [33] which are mainly expressed in the chicken oviduct and expressed abundantly in GE of cancerous ovaries from laying hens. Similarly, we found that expression of SERPINB12 mRNA and protein increase significantly in GE of in cancerous, but not normal ovaries of laying hens. As a matter of fact, among clade B serpins, SERPINB3 and SERPINB5 are well studied by various cancer researchers. SERPINB3 was first discovered in squamous cell carcinoma of the cervix [34] and it has been investigated in a variety of squamous cell carcinoma types [35], [36], [37], [38]. SERPINB3 is also involved in invasiveness of cells, as well as cell migration, apoptosis, and immune reaction [39], [40]. SERPINB5, also known as maspin (mammary serine protease inhibitor) is a class II tumor suppressor gene identified in patients with breast cancer [41]. SERPINB5 promotes apoptotic events of invasive prostate and breast carcinoma cells [41] and down-regulation of its expression causes pancreatic, colorectal, and ovarian adenocarcinomas [42]. On the contrary, over-expression of SERPINB5 was detected in two ovarian cancer cell lines including OVCAR3 and SKOV3 whereas the normal ovarian surface epithelial cells do not express SERPINB5 [43]. In our previous studies, we found increased expression of SERPINB3 in chicken ovarian carcinogenesis and suggested that it as a novel biomarker for predicting platinum resistance and poor prognosis for survival in patients with EOC [31]. In addition, SERPINB11 expression is dramatically increased in laying hens with progressive endometrioid adenocarcinoma of the ovary [30]. Furthermore, estrogen is a well-known risk factor for gynecological cancer in women [44]. Our previous report showed that *ESR1* mRNA expression is up-regulated 51-fold (*P*<0.001) in ovarian cancer in laying hens while there is little or no detectable ESR1 protein in normal ovaries [12]. Results of the present study indicate that expression of *SERPINB12* mRNA increased about 206-fold (*P*<0.001) only in ovarian carcinoma of laying hens. In addition, we observed that SERPINB12 localized predominantly to GE of cancerous ovaries. This glandular architecture is one of the most common characteristics of ovarian cancer in laying hens and women and it is estrogen-dependent and mainly appears in pre- and peri-menopausal women [26], [45]. Therefore, based on our current results, SERPINB12 may play an important role(s) in the development of EOC in laying hens, and be a potential biomarker for early detection and monitoring of therapies used to treat EOC in women.

Collectively, results of the current study demonstrate that *SERPINB12* is a novel estrogen-stimulated gene expressed specifically in GE during development and differentiation of the chicken oviduct. In addition, SERPINB12 is clearly associated with and may be an essential regulatory factor in abnormal growth and dysfunction of ovarian carcinomas. Therefore, our study provides new insights into the use of SERPINB12 as an ethiological and pathogenetical biomarker for chicken ovarian carcinoma and its use as a biomarker for diagnosis and monitoring of the effectiveness of therapies for treatment of EOC in women.

## Materials and Methods

### Experimental Animals and Animal Care

The experimental use of chickens for this study was approved by the Animal Care and Use Committee of Korea University. All chickens were exposed to a light regimen of 15 h light and 9 h dark with *ad libitum* access to feed and water, and subjected to standard poultry husbandry guidelines.

### Tissue Samples

#### Study one

Following euthanasia of 1- to 2-year-old laying White Leghorn (WL) hens, tissue samples were collected from brain, heart, liver, kidney, small intestine, gizzard, muscle, ovary and oviduct (n = 5). Subsets of these samples were frozen or fixed in 4% paraformaldehyde for further analysis. Frozen tissue samples were cut into 5- to 7-mm pieces, frozen in liquid nitrogen vapor, and stored at −80°C. The other samples were cut into 10-mm pieces and fixed in fresh 4% paraformaldehyde in PBS (pH 7.4). After 24 h, fixed tissues were changed to 70% ethanol for 24 h and then dehydrated and embedded in Paraplast-Plus (Leica Microsystems, Wetzlar, Germany). Paraffin-embedded tissues were sectioned at 5 µm.

#### Study two

Female chicks were identified by PCR analysis using W chromosome-specific primer sets (F: 5′-CTA TGC CTA CCA CAT TCC TAT TTG C-3′ and R: 5′-AGC TGG ACT TCA GAC CAT CTT CT-3′). Treatment with DES and recovery of the oviduct were conducted as reported previously [8]. We implanted a 10 mg DES pellet in the abdominal region of 1-week-old female chicks to release the hormone for 10 days. The DES pellet was removed from all chicks for 10 days, and then a 10 mg daily dose was administered for 10 additional days. Five chicks were assigned to each treatment group. For control chicks, tissues were collected from the middle part of the undifferentiated neonatal oviduct. On the other hand, four segments including infundibulum, magnum, isthmus, and shell gland were collected from the differentiated oviduct of DES-treated neonatal chicks.

#### Study three

Molting of laying hens was induced by adding 20,000 ppm zinc to the diet to effectively reduce feed-intake and induce molting [46], [47]. Briefly, molting was induced by feeding hens a diet containing high zinc (mixed 252 g zinc oxide per 10 kg feed to achieve a final concentration of 20,000 ppm of zinc). Laying hens in the molting group completely ceased egg production within 12 days after feeding the high zinc-diet. The 35 laying hens (47-week-old) were divided into two groups; a molting-progressing group and a post-molting-progressing group, and kept in individual cages. The molting group was further divided into three subgroups based on the number of days of feeding the high zinc diet (normal feeding group, and 6 days and 12 days after onset of zinc feeding). The recrudescence (post-molting) group was divided into four subgroups based on the number of days from complete cessation of egg laying and initiation of feeding a normal commercial diet: 20, 25, 30 or 35 days after onset of zinc feeding or 8, 13, 18 or 23 days on normal feed after cessation of egg production and removal from the high zinc diet.

#### Study Four

In this study, a total of 136 laying hens (88 over 36 months of age and 48 over 24 months of age) which had stopped laying eggs were euthanized for collection of normal and cancerous ovaries. From that population of laying hens, we obtained cancerous ovarian tissue from 10 hens and normal ovarian tissues from 10 egg-laying hens of similar age. We evaluated tumor stage of 10 hens with cancerous ovaries according to characteristic features of chicken ovarian cancer [26], [30]. Three hens had stage III disease as ovarian tumor cells had metastasized to the gastrointestinal (GI) tract and liver surface with profuse ascites in the abdominal cavity. Five hens had tumor cells spread to distant organs including liver parenchyma, lung, GI tract and oviduct with profuse ascites, indicating stage IV disease. Two hens had stage I disease as tumors were limited to their ovaries. Epithelial ovarian cancers in chickens were classified based on their cellular subtypes and patterns of cellular differentiation with reference to ovarian malignant tumor types in humans [26].

### RNA Isolation

Total cellular RNA was isolated from frozen tissues (100 mg) using Trizol reagent (Invitrogen, Carlsbad, CA) according to the manufacturer's recommendations. First, the tissues were homogenized with 1 ml Trizol using a power homogenizer and 0.2 ml of chloroform was added to homogenized samples. Then, the tubes were shaken vigorously by hand for a few seconds. The samples were incubated at room temperature for 3 min and centrifugated at 12,000×g for 15 min at 4°C. The aqueous phase of the sample was placed in a new 1.5 ml tube and 0.5 ml of isopropanol was added. After incubating the mixture for 10 min at room temperature, the samples were centrifuged at 12,000×g for 10 min at 4°C. The resulting RNA pellet was washed using 75% ethanol and dried in air. Finally, the pellet was resuspended in RNase-free water.?The quantity and quality of total RNA was determined by spectrometry and denaturing agarose gel electrophoresis, respectively. The quantity and purity of RNA were measured using a Nanodrop 2000 (Thermo Scientific, DE, USA). We confirmed RNA purity in samples with a 260/280 nm ratio greater than 1.8.

### Semiquantitative RT-PCR analysis

The cDNA was synthesized from total cellular RNA (2 µg) using random hexamer (Invitrogen, Carlsbad, CA) and oligo (dT) primers and AccuPower RT PreMix (Bioneer, Daejeon, Korea). The cDNA was diluted (1∶10) in sterile water before use in PCR. For *SERPINB12*, the forward primer (5′-TCT TAA CCA CGA AGC GTT CC-3′) and reverse primer (5′-AAC TTG TGT TCC CAG GAT GC-3′) amplified a 333-bp product. For *GAPDH* (housekeeping gene), the forward primer (5′-CAC AGC CAC ACA GAA GAC GG-3′) and reverse primer (5′-CCA TCA AGT CCA CAA CAC GG-3′) amplified a 443-bp product. The procedures for generation of primers, PCR amplification and verification of their sequences were as described previously [48], [49]. PCR amplification was conducted using approximately 120 ng cDNA as follows: (1) 95°C for 3 min; (2) 95°C for 20 sec, 60°C for 40 sec and 72°C for 1 min for 35 cycles for *SERPINB12* and 32 cycles for *GAPDH*; and (3) 72°C for 10 min. After PCR, equal amounts of reaction product were analyzed using a 1% agarose gel, and PCR products were visualized using ethidium bromide staining. The amount of DNA present was quantified by measuring the intensity of light emitted from correctly sized bands under ultraviolet light using a Gel Doc XR^+^system with Image Lab software (Bio-Rad).

### Quantitative RT-PCR Analysis

Total RNA was extracted from each tissue sample using Trizol (Invitrogen) and purified using an RNeasy Mini Kit (Qiagen). Complementary DNA was synthesized using a Superscript III First-Strand Synthesis System (Invitrogen). Gene expression levels were determined using SYBR Green (Biotium, Hayward, CA, USA) and a StepOnePlus Real-Time PCR System (Applied Biosystems, Foster City, CA, USA). The *GAPDH* gene was analyzed simultaneously as a control and used for normalization to account for variation in loading. *SERPINB12* and *GAPDH* were analyzed in triplicate. Using the standard curve method, we determined the level of expression of the examined genes using the standard curves and C_T_ values, and normalized them based on *GAPDH* expression. For *SERPINB12*, the forward primer (5′-GGC TGG AAC AGA CTG AAA GC-3′) and reverse primer (5′-TGA GGT TAA AGG TGC CTT CG-3′) amplified a 129-bp product. For GAPDH, the forward primer (5′-CAG AAC ATC ATC CCA GCG TC-3′) and reverse primer (5′-GGC AGG TCA GGT CAA CAA CA-3′) amplified a 133-bp product. The PCR conditions were 94°C for 3 min, followed by 40 cycles at 95°C for 30 sec, 60°C for 30 sec, and 72°C for 30 sec using a melting curve program (increasing the temperature from 55°C to 95°C at 0.5°C per 10 sec) and continuous fluorescence measurement. ROX dye (Invitrogen) was used as a negative control for the fluorescence measurements. Sequence-specific products were identified by generating a melting curve in which the Ct value represented the cycle number at which a fluorescent signal was statistically greater than background, and relative gene expression was quantified using the 2^−ΔΔCt^ method [50]. The relative quantification of gene expression was normalized to the Ct value for the control oviduct.

### *In Situ* Hybridization Analysis

For hybridization probes, PCR products were generated from cDNA using the primers described for RT-PCR analysis. The products were extracted from the gel and cloned into the TOPO Vector (Invitrogen, Carlsbad, CA). After verification of the sequences, plasmids containing gene sequences were amplified with T7- and SP6-specific primers (T7:5′-TGT AAT ACG ACT CAC TAT AGG G-3′; SP6:5′-CTA TTT AGG TGA CAC TAT AGA AT-3′) then digoxigenin (DIG)-labeled RNA probes were transcribed using a DIG RNA labeling kit (Roche Applied Science, Indianapolis, IN). Tissues were collected and fixed in fresh 4% paraformaldehyde, embedded in paraffin and sectioned at 5 µm on APES-treated slides. The sections were then deparaffinized in xylene and rehydrated to diethylpyrocarbonate (DEPC)-treated water through a graded series of alcohol. The sections were treated with 1% Triton X-100 in PBS for 20 min, washed two times in DEPC-treated PBS and then digested with 5 µg/ml Proteinase K (Sigma) in TE buffer (100 mM Tris-HCl, 50 mM EDTA, pH 8.0) at 37°C. After post-fixation in 4% paraformaldehyde, sections were incubated twice for 5 min each in DEPC-treated PBS and incubated in TEA buffer (0.1 M triethanolamine) containing 0.25% (v/v) acetic anhydride. The sections were incubated in a prehybridization mixture containing 50% formamide and 4× standard saline citrate (SSC) for at least 10 min at room temperature. After prehybridization, the sections were incubated overnight at 42°C in a humidified chamber in a hybridization mixture containing 40% formamide, 4× SSC, 10% dextran sulfate sodium salt, 10 mM DTT, 1 mg/ml yeast tRNA, 1 mg/ml salmon sperm DNA, 0.02% Ficoll, 0.02% polyvinylpyrrolidone, 0.2 mg/ml RNase-free bovine serum albumin and denatured DIG-labeled cRNA probe. After hybridization, sections were washed for 15 min in 2× SSC at 37°C, 15 min in 1× SSC at 37°C, 30 min in NTE buffer (10 mM Tris, 500 mM NaCl and 1 mM EDTA) at 37°C and 30 min in 0.1× SSC at 37°C. After blocking with 2% normal sheep serum (Santa Cruz Biotechnology, Inc., Santa Cruz, CA), the sections were incubated overnight with sheep anti-DIG antibody conjugated to alkaline phosphatase (Roche, Indianapolis, IN). The signal was visualized following exposure to a solution containing 0.4 mM 5-bromo-4-chloro-3-indolyl phosphate, 0.4 mM nitroblue tetrazolium, and 2 mM levamisole (Sigma Chemical Co., St. Louis, MO).

### Immunohistochemistry

Immunocytochemical localization of SERPINB12 protein was performed as described previously using a rabbit polyclonal antibody to SERPINB12 (catalog number: sc-85145; Santa Cruz Biotechnology, Inc., Santa Cruz, CA) at a final dilution of 1∶250 (0.4 µg/ml). Antigen retrieval was performed using the boiling citrate method as described previously [51]. Negative controls included substitution of the primary antibody with purified non-immune rabbit IgG at the same final concentration.

### Statistical Analyses

Data presented for quantitative PCR are expressed as mean ± SEM unless otherwise stated. Differences in variances between normal and cancerous ovaries were analyzed using the F test, and differences between means were subjected to the Student's t test. Differences with a probability value of *P*<0.05 were considered statistically significant. Excel (Microsoft, Redmond, WA) was used for statistical analyses.
